# TNT: An Interpretable Tree-Network-Tree Learning Framework using Knowledge Distillation

**DOI:** 10.3390/e22111203

**Published:** 2020-10-24

**Authors:** Jiawei Li, Yiming Li, Xingchun Xiang, Shu-Tao Xia, Siyi Dong, Yun Cai

**Affiliations:** 1 Tsinghua Shenzhen International Graduate School, Tsinghua University, Shenzhen 518055, China; li-ym18@mails.tsinghua.edu.cn (Y.L.); xxc17@mails.tsinghua.edu.cn (X.X.); 2 PCL Research Center of Networks and Communications, Peng Cheng Laboratory, Shenzhen 518055, China; 3 Ping An Life Insurance Company of China, Ltd., Shenzhen 518046, China; siyi.dsy.dong@gmail.com (S.D.); caiyun321@gmail.com (Y.C.)

**Keywords:** deep neural networks, James–Stein Decision Trees, distillable gradient boosted decision tree, interpretable machine learning, knowledge distillation

## Abstract

Deep Neural Networks (DNNs) usually work in an end-to-end manner. This makes the trained DNNs easy to use, but they remain an ambiguous decision process for every test case. Unfortunately, the interpretability of decisions is crucial in some scenarios, such as medical or financial data mining and decision-making. In this paper, we propose a Tree-Network-Tree (TNT) learning framework for explainable decision-making, where the knowledge is alternately transferred between the tree model and DNNs. Specifically, the proposed TNT learning framework exerts the advantages of different models at different stages: (1) a novel James–Stein Decision Tree (JSDT) is proposed to generate better knowledge representations for DNNs, especially when the input data are in low-frequency or low-quality; (2) the DNNs output high-performing prediction result from the knowledge embedding inputs and behave as a teacher model for the following tree model; and (3) a novel distillable Gradient Boosted Decision Tree (dGBDT) is proposed to learn interpretable trees from the soft labels and make a comparable prediction as DNNs do. Extensive experiments on various machine learning tasks demonstrated the effectiveness of the proposed method.

## 1. Introduction

Deep Neural Networks (DNNs) have achieved great success in many multimodal prediction tasks such as cross-modal embedding [1], image caption [2], and visual question answering [3]. However, as typical end-to-end models, DNNs usually work in a black-box paradigm [4,5] and the decision process is unknown for the test case, which limits the application of DNNs for some scenarios requiring explanation, such as medical or financial data mining and decision-making [6,7]. Besides, in some medical and financial problems, since data acquisition is susceptible to uncontrollable factors, the input data are sometimes low frequency and low quality. However, the learning process of DNNs usually require high-frequency and high-quality input data, and they easily overfit the training dataset [8], which also limits the application of DNNs.

As another kind of widely used model, the decision trees and tree-based ensemble models such as random forest or GBDT usually achieve better performance than other transitional machine learning algorithms. This observation is from counting the winning models of many big data competitions (www.kaggle.com). Although the performance is relatively good, the tree model has a simple basic structure and can be extended to a series of decision rules, thus it has intrinsic interpretability [5] for the test cases, especially when the depth of trees is not deep. Besides, the tree-based models have sophisticated tree nodes splitting strategy, therefore they are quite robust for processing low-frequency and low-quality data. Except for the traditional tree models, recently there are two new trends for designing the differentiable decision models. The first approach [9,10] is not limited to the tree shape and tries to construct a differentiable Directed Acyclic Graph (DAG), which has new loss function and learning modules. The other approach [11,12] leverages the knowledge distillation technique and uses differentiable soft decision trees as the base learner, thus it can be used for the student of a trained deep model.

**Figure 1:**
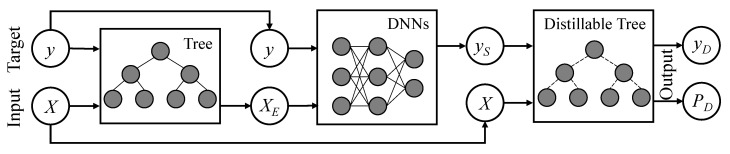
The workflow of our proposed Tree-Network-Tree (TNT) learning framework. Given the input data *X* and prediction target *y*, TNT first trains a tree-based model (e.g., random forest, GBDT, or our proposed JSDT) on the training dataset {X,y} and extracts the decision path of all trees to form an embedding representation $X_{E}$. Then, a deep model (e.g., DNN, CNN, or TCN) is trained on the embedding dataset $\left\{X_{E},y\right\}$ and generates the new soft labels $y_{S}$. Finally, a distillable tree model (e.g., soft decision trees, sGBM, or our proposed dGBDT) is trained on the soft label dataset $\left\{X,y_{S}\right\}$ and outputs the prediction value $y_{D}$ and the corresponding decision paths $P_{D}$. In general, the advantages of TNT come from three parts: the first tree model is robust for representing the dark knowledge in input data; the DNN model ensures good prediction performance; and the decision paths can be explicitly extracted from a distillable tree, therefore it is interpretable for decision-making.

**Figure 2:**
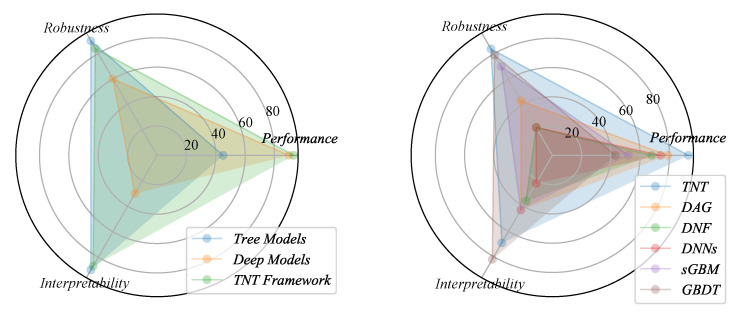
Comparisons among the deep models, tree models, and the proposed TNT framework.

In this paper, we propose a Tree-Network-Tree (TNT) learning framework, which is the integrated use of the tree models and deep learning techniques. As shown in Figure 1, our key is to introduce two tree models to improve the input and explain the output of DNNs. At the input end, we train the traditional tree models on the training data to obtain the decision rules to be the embedding representation [13]. Besides, we further propose a novel James–Stein Decision Tree (JSDT) to learn a preferable knowledge embedding. At the output end, we aim to introduce the interpretability for the test cases, but still keep a comparable prediction as to the deep models. Thus, the knowledge distillation technique is adopted to transfer the learned dark knowledge from the DNNs to the differentiable tree model, such as a novel distillable Gradient Boosted Decision Tree (dGBDT). As a result, our proposed TNT framework benefits from the advantages of different modules. Thus, it is robust for data, interpretable for output, yet still has high performance compared to the original deep model. An intuitive comparison is shown in Figure 2.

Based on the proposed TNT framework, we further explored the different ways of implementation, including the choices of data flow, and the potential end-to-end differentiable structures. We evaluated all these possible models on various machine learning tasks and conducted extensive experiments to show the interpretability of TNT for the medical diagnosis scenarios. In general, the main contributions of this paper are threefold:

- We improve the traditional decision tree and propose a novel James–Stein Decision Tree (JSDT) to provide better embedding representation of leaf nodes, which is more robust for the input data and applicable for DNNs.
- Inspired by recent advances on the differentiable models, we propose a distillable Gradient Boosted Decision Tree (dGBDT), which could learn the dark knowledge from DNNs and has interpretability for the test cases.
- To simultaneously improve the robustness and interpretability of the deep models, we explore potential pipelines, data flows, and structures on leveraging the tree models. Based on the analysis, we propose the TNT framework and verify it with extensive experiments.

## 2. Related Works

### 2.1. Deep Models in Black Box

Although the deep learning algorithms have achieved great success on various prediction tasks, they still suffer from lacking the robustness on input data and the interpretability for testing cases. Since most of the deep learning models learn weighting parameters with back-propagation and end-to-end mechanism [14], they are usually easy to be influenced by the data quality. However, for real-life applications, the collected data usually contain noise or even missing values.

In practice, collecting the dataset is usually subjective and the noise is easily induced, thus leading to a degradation of model performance. At the phase of model training, the noise could be in the feature representation or labels of the data. When the noise is randomly distributed in the feature dimension, it requires a sparsity-aware algorithm to tackle the sparse data, outliers, or missing values [15,16]. However, DNNs usually learn the weighting parameters for a fixed structure, thus lacking the ability of sparsity-awareness. From another point of view, to tackle the noisy labels, current DNNs usually require some noise adaption modules and a corresponding expectation-maximization (EM) optimization [17]. Except for the training noise, recent trends pay more attention to the deploying robustness, which requires no access to the gradient of the underlying DNNs to find adversarial examples [18] and attack the deployed deep models [19,20]. To defend against this kind of black-box attack and the adversarial examples, there could be a trade-off between the robustness and performance [21]. From the above observations, the DNNs suffer from the noise and variety of data, especially when the data are low frequency or low quality.

Besides, as the DNN model usually has a predetermined structure (e.g., the number of layers and neurons), the main purpose is to learn the weighting parameters. However, we cannot understand the model by just looking at those parameters, thus leaving a black-box system for making decisions [5]. Specifically, the parameters of CNNs are usually in two- or three-dimension filters and can be learned layer-by-layer [22]. There could be some spatial and temporal semantic information from visualizing the filters [14,23], but it is pretty hard to find out those effective filters, especially when the model is deep and has too many filters. According to the structure of input data, recently there are also many other deep convolutional models for various prediction tasks. The one-dimension CNNs [24] have one-dimension filters to capture the relationship among the very adjacent data points. Besides, the temporal convolutional networks (TCNs) are the state-of-the-art model for many financial sequence modeling tasks [25], but it has the one-dimension convolution, dilated convolution, and residual connection, making the prediction hard to understand.

### 2.2. Tree Models

According to the differentiability, the decision tree models can be divided into hard decision trees and soft decision trees. The traditional hard tree models are robust for data, while the novel soft tree models can be used for improving the interpretability of the deep models.

Different from the deep models, the traditional hard decision trees do not couple the weighting parameters and minimize a loss function to learn the model structure [5]. For the applications on tabular or structured data, we can adopt an ensemble of decision trees, such as the random forest [26], Gradient Boosted Decision Trees (GBDT), or Gradient Boosting Machine (GBM) [27], to learn the knowledge patterns. These tree models are quite robust for many real-world problems in two aspects. Firstly, it is quite common for the data to be noisy or ambiguous. A recent study [28] shows that the robust node splitting strategy could be very important for defending the adversarial examples. Secondly, limited by the collection process, the data pattern might be quite sparse or low frequency. For this situation, recent studies also show the potentiality of improving the robustness with the theoretical approach for both the decision trees or the tree ensemble [29,30,31].

Although these ensemble models consist of many subtrees, all of them can be extended to the decision rules and form a knowledge embedding of the training dataset [13]. Compared with the other deep embedding methods [32], tree-based embedding makes the feature representation directly interpretable, thus is highly suitable for building an explainable machine learning system [13]. Besides, while recent studies try to explore differential tree models [12,33,34], using the combination of neural networks and differential tree models is also a big trend: neural decision forests [35] use randomized multilayer perceptrons to learn the data-specific representations and find optimal predictions for the emerging child nodes. Deep neural decision forests [9] learn the feature representation from deep convolutional networks and have a differentiable decision forest to make the discrimination tasks. Another study explores the continuum of hybrid model in-between the decision forest and the convolutional neural networks and then proposes a directed acyclical fraphs [10] model. From the reported experiment of these studies, the differential tree models can achieve comparable performance than the state-of-the-art deep models.

### 2.3. Knowledge Distillation

In this paper, we adopt the knowledge distillation technique to make the deep models interpretable. The original usage of knowledge distillation is for compressing a deep model or ensemble model [36,37,38], which leverages a teacher–student paradigm to transfer the knowledge from a big model to a smaller one. Specifically, it utilizes a temperature function to distillate the soft labels, which are the softened logit values from the last fully connected layer of the teacher model, to replace the original labels to be the predicting target of the student model.

Beyond the standard approach, recent studies [39,40,41] show that knowledge distillation with soft labels can be beneficial for multiple ways and has been widely used. One of the new advances adopts the knowledge distillation technique for interpretable deep learning. Because knowledge distillation requires back-propagation operation on the student model, a study [11] creates an explainable network-tree learning framework, by using the differential property of the Soft Decision Tree (SDT) [33]. Because the soft decision tree follows a complete binary tree structure, every test case has a probabilistic decision path, thus implying a certain measure of interpretability. A shortcoming of distilling the knowledge from DNNs to a soft decision tree is that the capacity of the student model may limit the performance. To address this problem, another study [6] uses GBDT instead of SDT as the student model. However, since the adopted GBDT is an ensemble of the hard decision trees, it is still not differential and cannot leverage all the advantages of knowledge distillation in an end-to-end network-tree learning paradigm. In this paper, we extend the original GBDT [27] to a differential version and name it dGBDT, which can capture the distilled knowledge in a more coherent way, while still having interpretability for the test cases.

## 3. Proposed Tree Models

In this paper, we focus on improving the robustness and interpretability of the deep models for tabular and structured data. To achieve this, we adopt both the hard decision trees (e.g., JSDT) to process the input data and the soft decision trees (e.g., dGBDT) to explain the test cases.

### 3.1. James–Stein Decision Trees

CART [42] decision trees and its ensemble extension (e.g., random forests [26]) are widely applicable for both the classification and regression tasks. The usage of CART includes two stages: the tree construction and prediction. For the construction stage, most important is judging whether the division of a node is optimal, thus we need to calculate the information gain of the node before and after the division. Specifically, if a certain set of feature-values are used for splitting, and the information gain of the two sub-nodes obtained is the biggest, the division of node data brought by this set of feature-values is optimal.

Without loss of generality, we consider applying CART to a regression task. Assuming that the current dataset that needs to be divided is $D_{0}$, use the feature *a* and the corresponding value *v* to divide the data into two subsets $D_{1}$ and $D_{2}$. Then, we can find the optimal splits by minimizing the following loss function:

(1) $$\begin{array}{c} \min_{a,v}\left[\min_{c_{1}}\sum_{x_{i}\in D_{1}}{\left(y_{i}-c_{1}\right)}^{2}+\min_{c_{2}}\sum_{x_{i}\in D_{2}}{\left(y_{i}-c_{2}\right)}^{2}\right] \end{array}$$

where the values $c_{1}$ and $c_{2}$ are the optimal representation value of $D_{1}$ and $D_{2}$, respectively. When only considering the mean square error of a single subset $D_{j}$, the estimated value of its optimal value $\hat{c_{j}}$ is the simple average of all samples $x_{i}$ in the subset and has a representation value $y_{i}$, which can be denoted as:

(2) $$\begin{array}{c} \hat{c_{j}}=avg\left(y_{i}|x_{i}\in D_{j}\right) \end{array}$$

To construct a CART tree, the data of the root node can be injected into two sub-nodes, and then this process is repeated until the stop condition is met. Commonly used stopping conditions include that the tree reaches a maximum depth, the feature set is empty, and the number of samples of the node reaches the minimum value or is less than the minimum required number for the node to continue splitting. For the prediction stage of CART (or the corresponding random forest) algorithms, once all the trees are built and the predicted value of all leaf nodes are known, we take the simple average method to make the prediction.

The node splitting strategy in Formula (1) is quite clear but has a shortcoming. This strategy divides the feature space into multiple sub-spaces by learning training samples, which can get a high accuracy rate on the training set, but a too fine division might lead to serious overfitting, thus cause a reduced prediction result for new samples. In this paper, we propose the James–Stein Decision Tree (JSDT) to solve this problem. Different from the existing regression trees, which only consider the information of a single leaf node when predicting, the JSDT considers both the local data information of a single leaf node and the global data information contained in all leaf nodes [43]. Here, we present the generalization error of the JSDT and describe the concrete implementation in the following.

We first review the definition of James–Stein estimator. Assume *Y* is a *m*-dimension m≥4 random variable, which follows the multivariate Gaussian distribution and has an unknown mean μ and a known co-variance matrix $\delta^{2}I$, where $Y∼N(\mu ,\delta^{2}I)$. Now, we need to estimate a value $\hat{\mu }$ of the mean μ from *n* observed samples *y* from *Y*. Assuming that ν is an arbitrary fixed *m*-dimensional vector, then there is a James–Stein estimator [44] of the mean of *Y* that is a shrinkage estimator [45] of ν:

(3) $$\begin{array}{c} {\hat{\mu }}_{JS}=\left(1-\frac{\left(m-3\right)\sigma^{2}}{∥\bar{y}{-\nu ∥}^{2}}\right)\left(\bar{y}-\nu \right)+\nu ,m\geq 4 \end{array}$$

where $\bar{y}$ is the average value of *m*-dimensional samples. Using ${\hat{\mu }}_{MLE}$ to represent the average value obtained by maximum likelihood estimation, there is a proved comparison [46]:

(4) $$\begin{array}{c} L\left(\mu ,{\hat{\mu }}_{JS}\right)=E∥\mu -{\hat{\mu }}_{JS}{∥}^{2}<L\left(\mu ,{\hat{\mu }}_{MLE}\right)=E{∥\mu -{\hat{\mu }}_{MLE}∥}^{2},m\geq 4 \end{array}$$

which means the mean square loss caused by the James–Stein estimator is smaller than the maximum likelihood estimation, when the feature dimension of data is larger than 4. In this paper, we consider the regression tree with a binary structure, thus the James–Stein estimator cannot be directly applied to the node splitting process. To improve the hard tree on both the construction and prediction stages, we propose a new feature selection method by leveraging both the James–Stein estimator and maximum likelihood estimator, and list this process in Algorithm 1.

Before splitting the node *N*, we denote the temporary completed tree with $g_{temp}$, and denote the number of leaf nodes with $m_{temp}$. Then, we split the node *N* into the sub-nodes $N^{\prime }$ and $N^{\prime \prime }$ by using the feature *a* and its value *v*, and update the tree as $g_{temp}^{\prime }$. At this point, the sub-nodes $N^{\prime }$ and $N^{\prime \prime }$ are the new leaf nodes of tree $g_{temp}^{\prime }$. If the condition $m_{temp}\geq 4$ is meet, we update the mean value of all the leaf nodes with the James–Stein estimator. Otherwise, we adopt the maximum likelihood estimator to update the mean value of the leaf nodes $N^{\prime }$ and $N^{\prime \prime }$, then finding the best split feature and the corresponding value ${\left(a,v\right)}_{best}$ with Formula (1). Iterate this process until the tree is built.
**Algorithm 1** Feature selection of James–Stein Decision Tree (JSDT).**Input**: Current node *N*, the feature sets (A,V), the number of leaf nodes $m_{temp}$, and the stop condition.**Output**: The best split feature ${\left(a,v\right)}_{best}$.1:**if** Node *N* meets the stop condition **then**2: Label current node *N* as the leaf node; **return**3:**end if**4:Initialize current split loss with $L_{min}=\infty$, and the split feature ${\left(a,v\right)}_{best}$.5:**for** Every feature (a,v) on the feature set (A,V) **do**6: Split the node *N* into two sub-nodes $N^{\prime }$ and $N^{\prime \prime }$;7: **if** $m_{temp}\leq 3$ **then**
8:  Calculate the information gain and the mean value of nodes $N^{\prime }$ and $N^{\prime \prime }$;9: **else**
10:  Calculate the mean value on nodes $N^{\prime }$ and $N^{\prime \prime }$ with the simple average.11: **end if**
12: Calculate the sum of mean square loss $L_{temp}$ on nodes $N^{\prime }$ and $N^{\prime \prime }$.13: **if** $L_{temp}<L_{min}$ **then**
14:  $L_{temp}=L_{min}$, ${\left(a,v\right)}_{best}=\left(a,v\right)$;15: **end if**
16:**end for**17:**return** The best split feature ${\left(a,v\right)}_{best}$.

When the variance is unknown, to ensure the weights of global information and local information are both positive, we usually adopt a variant of Formula (3) [47,48], which is:

(5) $$\begin{array}{c} {\hat{\mu }}_{i}^{JS+}=GM+{\left(1-\lambda \cdot \gamma \right)}_{+}\cdot \left({\tilde{y}}_{i}-GM\right),m\geq 4 \end{array}$$

where the $GM=\frac{1}{m}\sum_{i=1}^{m}{\tilde{y}}_{i}$ is the global mean, ${\tilde{y}}_{i}$ is the simple average of the samples on the leaf node $N_{i}$ (also is the local mean), and $\gamma =\left(m-3\right){\left(\sum_{i=1}^{m}\frac{n}{\delta_{i}^{2}}{\left({\tilde{y}}_{i}-GM\right)}^{2}\right)}^{-1}$ is a shrinkage factor, with $\delta_{i}^{2}$ the variance on leaf node $N_{i}$ and ${\left(1-\gamma \right)}_{+}=max\left(0,1-\gamma \right)$. Due to the value of γ is determined by the data distribution and might be too small to change the splitting, we also introduce a scale parameter λ.

In summary, the tree models (including JSDT) are robust for input data in the following aspects: (1) they can directly process almost all feature types, no matter the data are numerical or categorical; (2) they can process the samples with missing values for features and do not need to discard these data; and (3) the features are not required to correlate with each other, and the unrelated features can also be used to construct the decision tree. Besides, JSDT further considers the relationship among all the samples on leaf nodes, which could shrinkage leaf values and relieve the overfitting.

### 3.2. Distillable Gradient Boosted Decision Trees

Before presenting details on the proposed dGBDT, we first give brief introduction on the background information about GBM, GBDT [27], SDT [33], and soft Gradient Boosting Machine (sGBM) [12] models.

Without loss of generality, we consider the regression scenario. Given a training dataset ${\left\{x^{i},y^{i}\right\}}_{i=1}^{N}$, the goal of GBM is to train an ensemble of *m* sub-trees, in which the output $F_{m}\left(x\right)$ of *m*th tree approximates the accumulated error $\sum_{i=1}^{N}\left[y_{i}-F_{m-1}\left(x_{i}\right)\right]$. As a widely used implementation of GBM, GBDT implies more concrete to the boosting strategy and also has the additive ensemble loss $F\left(x\right)=\sum_{m=0}^{M}\beta_{m}F_{m}\left(x;\theta_{m}\right)$, where the $\theta_{m}$ is the parameters of the *m*th tree and $\beta_{m}$ is the weighting coefficient. Beyond GBDT, on the one hand, recent studies [15,49] leverage many strategies (e.g., the regularization terms for the tree complexity and loss function, pruning, and shrinkage estimation) to further improve the hard boosted trees. On the other hand, some studies [12,33,34] try to explore differential tree models. SDT [33] uses a probability calculated sigmoid gating function $g_{m}\left(x\right)$ to learn the soft decision nodes, and estimate the posterior probability of the left and right children: $P\left(L|x\right)=g_{m}\left(x\right)$ and $P\left(R|x\right)=1-g_{m}\left(x\right)$. Since the prediction output for input sample is the weighted sum of class distributions among all leaf nodes, where the weight is the product of the cumulative probability on internal nodes along the decision paths, SDT is differentiable and can be trained via back-propagation. Using SDT as the base learner, the corresponding sGBM [12] has significant advantages over GBDT. First, sGBM is differentiable and has learnable parameters, so the model can be updated online by the low-cost fine-tuned training. Then, benefitting from the mini-batch gradient descent, sGBM can better train all the trees simultaneously and support the multi-output regression tasks, and hence is more efficient.

The soft trees can achieve more efficient training and comparable performance on the regression tasks than the hard trees [12,33]. However, while the deep models usually achieve a better performance, recent proposed N-T method [11] uses the knowledge distillation [36] to transfer the power of DNNs into soft trees. They train the SDT for classification by minimizing the cross-entropy between each leaf and the target distribution with the loss function: $L\left(x\right)=-\log(\sum_{l}P^{l}\left(x\right)\sum_{k}T_{k}\log Q_{k}^{l})$, where $Q^{l}$ is the learned probability distribution at the leaf node *l*, $P^{l}\left(x\right)$ is the probability of arriving the leaf node *l*, and *T* is the soft target distribution from the pre-trained DNNs. In general, to implement the knowledge distillation from DNNs to the tree models, it usually requires the twice labeling technique. As for a typical regression task, the basic loss function of knowledge distillation could be $L\left(x\right)=\alpha T^{2}MSE\left(O_{s}-O_{t}\right)+\left(1-\alpha \right)MSE\left(O_{s}-y\right)$, where *y* denotes the original label, while $O_{s}$ and $O_{t}$ are the output value of the student and teacher model, and α and *T* are the trade-off weighting and distillation temperature, respectively.

**Figure 3:**
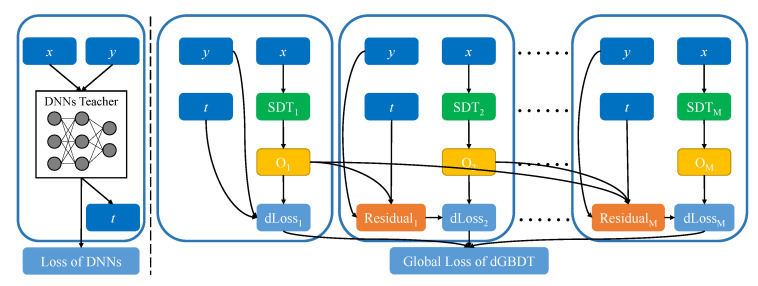
Data flow of the proposed Distillable Gradient Boosted Decision Trees (dGBDT): (**Left**) obtain the soft label *t* from the teacher DNNs model; and (**Right**) train the dGBDT.

Concretely, assume we have *M* different SDT [33] to be the base learners, which are denoted as ${\left\{h_{m}\right\}}_{m=1}^{M}$, parameterized with $\theta_{m}$ and have output $o_{m}$. Then, the cumulative output of the learned dGBDT is $s_{m-1}^{i}=\sum_{j=0}^{m-1}o_{j}^{i}\left(x^{i},\theta_{j}\right)$. For the training phase, a global loss for all the trees is defined as $L=\sum_{m-1}^{M}l_{m}$, where $l_{m}$ is the loss for each SDT, which is defined with a MSE loss $l_{m}={∥r_{m}-o_{m}∥}_{2}^{2}$. $r_{m}$ is the corresponding residual calculated by a distillation loss:

(6) $$dl\left(s_{m-1}^{i},\alpha ,y^{i},t^{i}\right)=\alpha T^{2}∥s_{m-1}^{i}-t^{i}{∥}^{2}+\left(1-\alpha \right){∥s_{m-1}^{i}-y^{i}∥}^{2}\;$$

where the formulation is calculated on the sample $\left(x^{i},y^{i}\right)$, in which $y^{i}$ denotes the label value, while $t^{i}$ denotes the soft label generated by the DNNs. Then, α and *T* are the weighting and temperature parameters of the distillation, respectively. We show the training of dGBDT in Algorithm 2 and illustrate the data flow of the proposed dGBDT model in Figure 3.
**Algorithm 2** Training Distillable Gradient Boosted Decision Trees (dGBDT).**Input**: Training batches $B=B_{1},B_{2},\ldots ,B_{\left|B\right|}$, number of trees *M*, dGBDT parameters $\theta ={\left\{\theta_{m}\right\}}_{m=1}^{M}$.**Output**: The updated dGBDT parameters θ.1:**for**b=1 to |B| **do**2: Initialize the output $o_{0}^{i}\leftarrow 0$ of the first tree for $x^{i}\in B_{b}$;3: **for** m=1 to *M* **do**4:  Infer the output $o_{m}^{i}\leftarrow h_{m}\left(x^{i};\theta_{m}\right)$ on current tree for $x^{i}\in B_{b}$;5:  Calculate the sum of past outputs $s_{m-1}^{i}=\sum_{j=0}^{m-1}o_{j}^{i}$ for $x^{i}\in B_{b}$;6:  Find the residual term from distillation loss $r_{m}^{i}\leftarrow -\partial \left[dl\left(s_{m-1}^{i},\alpha ,y^{i},t^{i}\right)\right]/\partial \left[s_{m-1}^{i}\right]$ for $x^{i}\in B_{b}$;7:  Record the loss of current tree $l_{m}\leftarrow \sum_{x^{i}\in B_{b}}{∥r_{m}^{i}-o_{m}^{i}∥}_{2}^{2}$ for $x^{i}\in B_{b}$;8: **end for**
9: Update θ w.r.t. the global loss $L\leftarrow \sum_{i=1}^{M}l_{m}$ using gradient decent;10:**end for**11:**return** The trained model parameter θ of dGBDT.

From the view of model structure, the proposed dGBDT can be regarded as a specific implementation of sGBM [12]. While sGBM [12] only assumes the basic learners to be differentiable, our proposed dGBDT further specifies SDT [33] to be the basic tree learners and has two important differences from sGBM. First, the parameters of dGBDT are optimized from an explicit distillation loss, which is different from the ground truth loss in sGBM. In this way, each basic SDT of dGBDT is trained with the implicated dark knowledge of the DNNs teacher, thus can be more powerful and flexible. Second, The data flow in both dGBDT and SDT follows a loop-free DAG structure, thus the parameters can be optimized via back-propagation and the deployed decision rules are soft. In this way, for any test case, the feature of data can be used for the explanation of decision-making.

## 4. Proposed TNT Framework

In this paper, we explore the potential pipelines and structures on leveraging the tree models to improve the deep models. As a result, we propose the TNT framework to simultaneously improve the input robustness and interpretability of the DNNs. Based on TNT, we also discuss the variants for different kinds of data flows, which relies on the knowledge embedding and distillation techniques.

### 4.1. Tree-Network-Tree Learning Framework

While deep models achieve state-of-the-art performance on various applications, the tree models are known to be robust at the training phase and can be expended to interpretable decision rules at the deploy phase. A series of studies [9,10,32,35,50,51] claims to propose a better learning framework by leveraging the advantages of these two models. In Table 1, we present a superiority analysis on the possible workflows of algorithm pipelines and corresponding data flows. While the existing methods work in the “T-N” and “N-T” patterns, our proposed “T-N-T” framework could leverage the advantages of the tree and deep models to the maximum potential.

To improve the robustness of the deep models for the tabular and structured data, an effective way is to learn the DNNs from a tree embedding [32,50,51] and work in a “T-N” learning pattern. In general, we can repeat two steps to learn a decision tree: select the feature and split the nodes. In a straight-forward way, we can optimize a cross-entropy loss on the one-hot embedding of all leaf nodes for DNNs to learn the dark knowledge. However, if we adopt a tree ensemble (e.g., GBDT) to capture the knowledge embedding, the number of leaf nodes will have a sharp increase, thus lowering the efficiency. A recent approach [50] adopts the *leaf embedding* and *tree grouping* techniques to ease this problem.

The *leaf embedding* strategy relies on a learnable mapping function $X_{E}=H\left(L_{t}\left(X\right);w_{t}\right)$. For a single tree *t*, it learns parameter $w_{t}$ to map the original one-hot leaf index $L_{t}\left(x\right)$ of samples *X* to the dense embedding $X_{E}$. Then, DNNs take the embedding $X_{E}$ as input to learn the parameter $w_{n}$ by minimizing $E_{X}\left[l_{1}\left(w_{n}X_{E},p_{t}\left(X\right)\right)\right]$, where $p_{t}\left(X\right)$ denotes the predict leaf value of sample. The leaf embedding strategy avoids representing all the leaf values with a sparse one-hot vector. It is more useful for the tree ensemble, because the number of leaf nodes increases linearly with the increase of the number of trees. The *tree grouping* strategy takes equally randomly grouping on all the trees of a tree ensemble. For a group of trees *T*, it concatenates all the leaf nodes into $L_{T}\left(X\right)$ and utilizes the leaf embedding to learn DNNs by minimizing $E_{X}\left[l_{1}\left(w_{n}H\left(L_{T}\left(X\right);w_{t}\right),\sum_{t\in T}p_{t}\left(X\right)\right)\right]$.

Except for the *leaf embedding* and *tree grouping*, the “T-N” part in our “T-N-T” pattern relies on one additional *leaf shrinking* strategy. Specifically, we achieve this strategy with the proposed JSDT model as described in Section 3.1. For a regression task, JSDT shrinks leaf value $p_{t}^{JS}\left(X\right)$ to a smooth distribution and learn the DNN parameter $w_{n}^{JS}$ by minimizing $E_{X}\left[l_{1}\left(w_{n}^{JS}X_{E},p_{t}^{JS}\left(X\right)\right)\right]$. Considering the implementation of the “T-N” part, we set the input of DNNs to be the output of GBDT by default. However, due to any layers of DNNs that could be used for learning the embedding of GBDT, we also adopt a *joint training* strategy for the last layer of DNNs [32]. While GBDT can efficiently memorize the knowledge embedding of sparse feature interactions, DNNs can generalize to the unseen feature based on the observed embeddings.

Benefitting from the above-mentioned strategies, the “T-N” part of “T-N-T” can distill a tree ensemble (e.g., GBDT) into compact DNNs, which improves the robustness and performance.

To provide the interpretability for test cases, there are some implementations [9,10,11,12,52] following the “N-T” learning pattern. Specifically, the first kind of approaches [9,10] has a fully differentiable Directed Acyclic Graph (DAG) and learns all the model parameters at the same time. The default “N-T” part in our “T-N-T” pattern follows the second kind of approaches [11,12], which leverages the knowledge distillation technique and uses the dGBDT model as a student model.

### 4.2. Further Exploration

Without loss of generality, assume that we fit a TNT model on the dataset {X,y} to obtain the output $y_{D}$ and decision path $P_{D}$ for the test cases. Beyond the default TNT setting, in this paper, we also explore various data flow strategies and model candidates to cover the possible implementations of the proposed TNT framework.

**Figure 4:**
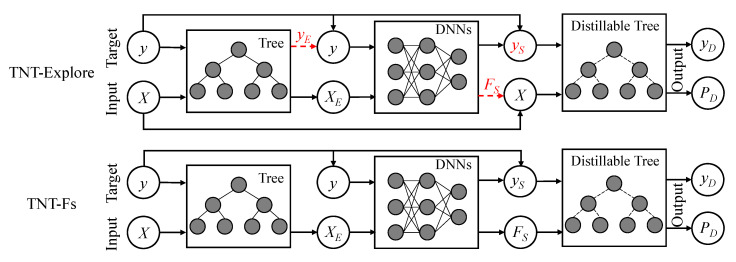
Based on the TNT learning framework, we further explore the different data flow strategies.

To find a preferable TNT structure, we consider the different data flow strategies and illustrate them with *TNT-Explore* in Figure 4. At first, we need to train the DNNs from the embedding $X_{E}$, so we fix the feature input and try different predicted target $y_{T}$, which is the tree prediction. However, limited by the model capacity, the predicted label of hard tree models usually captures more noise than the original label *y*, thus cannot be an alternative option. Therefore, we consider no changeable for the data flow in the “T-N” part. As for the “N-T” part, except for using the soft label *t* for the distillable tree, according to the distillation loss in Formula (6), we can also use a mixed label $y_{S}$, which is a combination of the ground truth *y* and the soft label *t*. Then, keeping up with the *joint learning* strategy [32], the feature input *X* could be replaced by the selected feature $F_{S}$ from different layers of DNNs. In such a situation, we transfer the distilled knowledge on the selected feature layers [39] and call the new structure as *TNT-Fs*.

**Figure 5:**
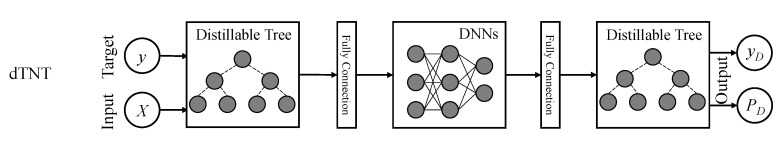
Based on the TNT framework, we further propose the fully differentiable TNT structure.

Following the previous approaches [9,10], we also consider a fully differential TNT, in which all the three parts could be optimized by the SGD algorithm. Specifically, we change the hard tree part of TNT to a distillable tree and call the new structure as *dTNT*. To normalize the data flow, we insert a fully connected layer between the adjacent models. The structure is illustrated in Figure 5.

## 5. Experiments

We conducted experiments on the proposed TNT, TNT-Fs, and dTNT structures to explore three questions: (1) Does the proposed TNT learning framework achieve comparable performance to the state-of-the-art methods? (2) Do the TNT approaches perform more robustly than other methods on tabular data? (3) Do the TNT approaches help decision-making and how can the prediction results be explained? To answer these questions, we conducted extensive experiments on various datasets, including both tabular data and image modals. A brief summary on the tabular datasets is given in Table 2.

### 5.1. Datasets and Setup

The **Cancer** refers to the UCI Wisconsin breast cancer dataset (https://archive.ics.uci.edu/ml/datasets/Breast+Cancer+Wisconsin+(Diagnostic)). The task is to diagnosis a breast tumor as benign or malignant from the extracted 30 different nuclear features. In our experiments, we changed the binary classification task to a regression task and predicted the risk probability (0 refers to benign, 1 refers to malignant). This dataset is small, thus is suitable for evaluating a low capacity implementation of TNT, which is formed by single tree model and shallow DNNs. All the feature values in these data are numeric and contain no missing entry. For each setup, we randomly split 80% instances as training set and used five-fold cross-validation for evaluating the models.

The **Criteo** refers to a kaggle challenge dataset (http://labs.criteo.com/2014/02/download-kaggle-display-advertising-challenge-dataset) and the task is to predict the click rate. Because the dataset is quite large and contains 51.8 million instances and 39 features, we used this dataset to evaluate the high capacity implementation of TNT, which is formed with the ensemble trees and the following “N-T” modules. As some features in the data have missing values, we trained the first “T” module for generating the leaf embedding, and then used the embedding for training the “N-T” modules. To generate stable leaf embedding, we followed the preprocess strategy in a previous study [15] and adopted the statistics of average CTR and count of ID features to replace the original values.

The **NASDAQ** dataset [53] collects the sequential data from the transaction records of 1026 stocks in the NASDAQ market between 2 January 2013 and 8 December 2017. We used the original train–valid–test split in the experiment and applied the TNT framework to make the relational stock ranking task. Because the dataset also includes topology relationship between the companies such as Wiki company-based relations, we also show how to find the clues of decision-making from distillable trees.

The **MIMIC-III** dataset (https://physionet.org/content/mimiciii/1.4) contains 38,425 hospital admissions of adult patients (aged 15 years or above) first admitted to an ICU. Limited by the data collection of medical monitoring, this dataset contains missing values. Thus, we followed the previous setting [54] to preprocess the data and extracted 22 different features to better measure the status of patient stay. We compare different methods on the mortality prediction task and show how the proposed TNT achieves interpretable decision-making.

To intuitively display the interpretable knowledge discovery, we also conducted experiments on a **CVOID-19 CT** image dataset (https://github.com/UCSD-AI4H/COVID-CT) for the task of medical diagnosis. This dataset consists of 349 CT scans that are positive for COVID-19 infection and 397 CT scans for patients not infected by COVID-19. We followed a previous setting [55] to split the dataset into training, validation, and test sets with the ratio 0.6:0.15:0.25. We fine-tuned a pre-trained DenseNet-169 to be the basic deep model.

### 5.2. Robustness and Performance

To evaluate and analyze the performance and robustness of the proposed TNT framework, we conducted extensive ablution studies for various tree models, deep models, the fusion of tree and DNNs, and the proposed TNT on four tabular datasets. The area under receiver operating characteristic curve (AUROC) and the area under the precision-recall curve (AUPRC) were the two adopted metrics.

As a baseline, we first tested trees, DNNs, the fusion of trees and networks, and the proposed TNT methods on the original Cancer dataset, which is not sparse. Then, we evaluated the model robustness on the sparse version of the Cancer dataset. The sparsity is caused by artificially and randomly wiping out certain percent (20% and 40% in the experiments) of the values and leaving the entries empty. As for the model parameters: (1) we searched and fixed the best shrinkage parameter λ=25 for the six-layer JSDT; (2) a isx-layer MLP (with 32-16-16-8-8 neurons in the hidden layers) was used as the DNNs; (3) the W & D model has 16 nodes for the wide part and a six-layer MLP (with **16**-16-16-8-8 neurons in the hidden layers) for the deep part; and (4) the adopted SDT [33] has six layers and trained via standard SGD, while the distillation followed a previous study [11]. Note that the first T & N fusion was configured as a Wide and Deep model [32] and trained in a typical DAG [10] manner.

We show the average AUROC and AUPRC values of five independent trials in Table 3. In general, to fit the Cancer dataset, all the adopted models are designed with low capacity, thus sensitive to the sparsity. However, from the result, the proposed TNT is quite robust among all the approaches, and the T-N fusion also achieves relatively good robustness. The ablation study about JSDT and CART also shows that leaf embedding is more robust than one-hot embedding.

**Figure 6:**
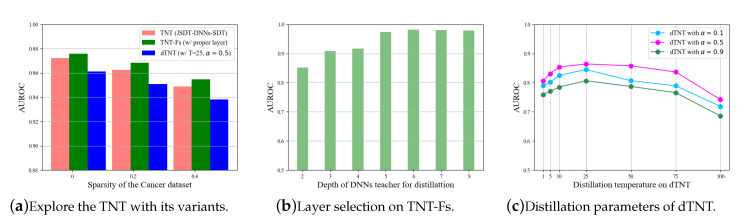
Further exploration of the TNT framework. The experiments on TNT-Fs and dTNT structures.

Except for the basic TNT framework, we further explored the model parameters based on the TNT-Fs and dTNT structures. The experiments were conducted on the Cancer dataset and the results are shown in Figure 6. While the DNNs in TNT are fixed to the six-layer MLP, we tried to extract the dark knowledge from different layers and formed the TNT-Fs structure, which could achieve better performance. We also explored the distillation parameters of the fully distillable dTNT structure.

We also conducted experiments on three larger datasets to evaluate model performance. To better capture the knowledge embedding from the original dataset, we extended the proposed JSDT into an ensemble version, which follows the random forest algorithm and named as the James–Stein’s Decision Forest (JSDF). The tree ensemble has 80 sub-trees and the tree depth was limited to less than 10. We implemented the dGBDT with SDT, while the sGBM was with CART. We used different deep models for different datasets: (1) in the Criteo experiment, we followed a previous study [56] to set the hyper-parameters and reproduced the DeepFM (denoted as DFM) and FM and DNN variant of W & D model; (2) for NASDAQ, we preprocessed the dataset to obtain a graph of the stock relations and trained a Rank_LSTM model (shortened to rLSTM) as described in a previous study [53]; and (3) for MIMIC-III, we followed a previous study [54] and fine-tuned the multi-scale ConvNet model (shortened to Conv) to be the baseline. Besides, we used the same distillation strategy [11] but searched for different trade-off weights for different N-T structures. To better evaluate the performance, we also adopted the Log Cross Entropy Loss (LogLoss), Mean Square Error (MSE), and Mean Reciprocal Rank (MRR) metrics for the Criteo and NASDAQ tasks, where smaller LogLoss (≥0), smaller MSE (≥0), and larger MRR ([0,1]) indicate better performance.

We repeated the experiments for five independent trails and show the averaged results in Table 4. From the observation of different tasks, all the deep learning models achieve better performance than the tree models. The best performance is achieved by the T & N fusions and especially the T-N patterns, which adopts the tree ensemble to handle various input types. The proposed TNT framework achieves comparable performance as the T-N models. From the ablation studies between different tree models (e.g., GBDT-[DNNs] vs. JSDF-[DNNs] and GBDT-[DNNs]-dGBDT vs. JSDF-[DNNs]-dGBDT), we found that the JSDF yields better knowledge embedding than the GBDT. Besides, the ablation studies on the GBDT-[DNNs]-sGBM and GBDT-[DNNs]-dGBDT show that soft tree ensemble could be a better student model for the distillation of deep models.

### 5.3. Interpretability

As shown in Table 4, the T-N fusion and the proposed TNT methods achieve comparable performance and outperform the original deep methods. However, because the final input of the T-N pattern is the tree embedding $X_{E}$, it is still hard to interpret the T-N fusion for decision-making. The TNT approaches, by contrast, have the tree model to be the final learner and take the original data feature *X* as input. Therefore, it is worth investigating TNT and figuring out how it helps decision-making. In the following, we first interpret the proposed TNT framework by presenting the partial dependence plots (PDPs) [5] between data features on the ICU mortality prediction task. Then, we visualize the Class Activation Mapping (CAM) [23] for the T-N fusion and TNT models on a CT image diagnosis task. Both tools provide a visualization for the interpretability of the decision-making of medical diagnosis.

#### 5.3.1. Partial Dependence Plots

The visualizations of partial dependence plots (PDPs) [5] intuitively show the relationships between the prediction output and features. Specifically, the PDPs are calculated by marginalizing the prediction value over the selected features. When the calculation is between the prediction and one single feature, the visualization is *one-way PDPs*; when the calculation is on the prediction and an interaction of two features, the visualization is *two-way PDPs*.

**Figure 7:**
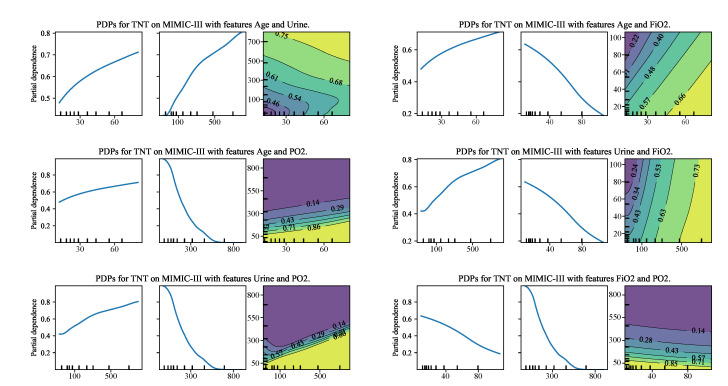
Partial dependence plots of the selected features from dGBDT for ICU mortality prediction tasks. Yellow denotes positive dependence and blue denotes negative dependence.

To draw the PDPs for the mortality prediction task, we fine-tuned the final dGBDT module of the TNT (JSDF-Conv-dGBDT) on a subset of the MIMIC-III dataset, which only contains 3-h of data for each patient. We analyzed the results and selected some of the one-way PDPs and corresponding two-way PDPs, as shown in Figure 7. The features such as the *Fraction of inspired oxygen (FIO2)* and *Oxygen pressure in blood (PO)* have negative correlations to the mortality rate, while *Age* and *Urine output* have positive relationship. These findings are clinically significant, which provide more insights into the results of the deep models and helpful for decision-making.

#### 5.3.2. Classification Activation Mapping

One way to evaluate the tree model is to compare its performance and interpret the fine-grained decision rules. However, because the tree model in the output end of the TNT framework is differentiable, we can also provide more intuitive visualization to interpret the prediction, such as drawing the Class Activation Mapping (CAM) [23] on a CT image. To obtain the final CAM of dGBDT, we regard each SDT as a following differentiable layer after the CNNs and aggregate all their heatmap responses.

**Figure 8:**
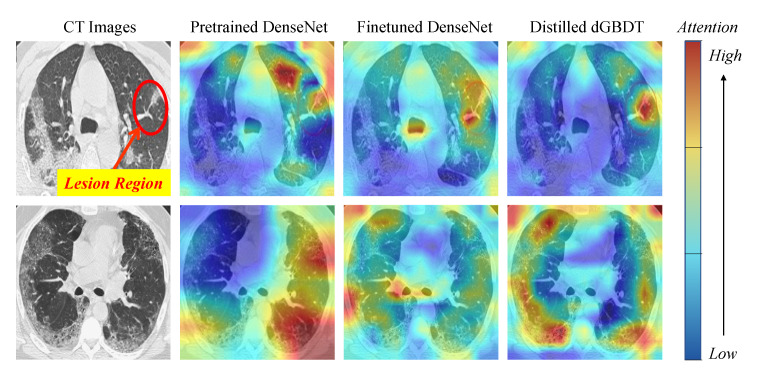
CAM visualizations for the ImageNet pre-trained DenseNet, the COVID-19 CT fine-tuned DenseNet, and the distilled dGBDT. The first row is an instance for a COVID-19 CT (in which the lesion region is labeled by a human doctor), while the second is for a Non-COVID-19 CT.

Because CT images do not require a hard tree model for the pre-processing, we degraded the TNT model to the N-T pattern and just distilled the knowledge from a pre-trained DenseNet-169 to obtain the following dGBDT. The CAM visualizations are shown in Figure 8. Comparing the responses of the DenseNet-169 and dGBDT, we notice that dGBDT pays more attention to some of the disease-related visual localization, thus improving the reliability of the prediction and diagnosis.

## 6. Conclusions

In this paper, we propose a Tree-Network-Tree (TNT) learning framework for explainable decision-making, where the knowledge is alternately transferred between the tree model and DNNs. In the input end, a novel James–Stein Decision Tree (JSDT) is proposed to generate better knowledge representations for DNNs. In the output end, a novel distillable Gradient Boosted Decision Tree (dGBDT) is proposed to learn interpretable trees from the soft labels and make a comparable prediction as DNNs do. Beyond the default setting, we also explore various data flow strategies and model candidates to cover the possible implementations of the proposed TNT framework. Extensive experiments on various machine learning tasks demonstrated the effectiveness of the proposed method. 

## Figures and Tables

**Table 1:** The analysis of superiority for different pipelines on tree and deep models. “*√*” represents good; “x” represents not good; “T” is tree model; and “N” is deep model.

	T	N	T-N	N-T	T-N-T	N-T-N	T-N-T-N	Others
Performance	x	*√*	*√*	*√*	*√*	*√*	makes sense	redundant
Robustness	*√*	x	*√*	x	*√*	x	but is	and not
Interpretability	*√*	x	x	*√*	*√*	x	redundant	necessary

**Table 2:** The task description for four tabular datasets. We also list the size number as Sample × Feature.

	Size	Task Description		Size	Task Description
Cancer	569 × 30	Risk Probability Prediction	NASDAQ	1026 × 1245	Relational Stock Ranking
Criteo	51.8 M × 39	Click Rate Prediction	MIMIC-III	38,425 × 22	ICU Mortality Prediction

**Table 3:** The **robustness** analysis on the Cancer dataset with different level of missing values. **Bold** indicates the minimal and the second minimal performance degradation.

Methods		Cancer (No Sparse)		Cancer (20% Sparse)		Cancer (40% Sparse)	
		AUROC	AUPRC	AUROC	AUPRC	AUROC	AUPRC
Tree Models	CART (single tree)	0.9367	0.9529	0.9273	0.9449	0.9114	0.9424
	JSDT (single tree)	0.9449	0.9561	0.9341	0.9496	0.9185	0.9480
Deep Models	DNNs (6-layer MLP)	0.9665	0.9522	0.9394	0.9428	0.9288	0.9227
T & N Fusion	W & D (DAG pattern)	0.9779	0.9496	0.9565	0.9423	0.9468	0.9312
	CART-DNNs (T-N)	0.9742	0.9463	0.9610	0.9428	0.9474	0.9357
	JSDT-DNNs (T-N)	0.9784	0.9531	0.9629	0.9487	0.9523	0.9398
	DNNs-SDT (N-T)	0.9620	0.9440	0.9381	0.9331	0.9223	0.9207
Proposed TNT	CART-DNNs-SDT	**0.9674**	0.9460	0.9602	0.9387	**0.9436**	0.9340
	JSDT-DNNs-SDT	**0.9723**	0.9471	0.9626	0.9406	**0.9488**	0.9389

**Table 4:** Evaluation of the performance on Criteo, NASDAQ, and MIMIC-III datasets. The [DNNs] refer to DeepFM (DFM), Rank_LSTM (rLSTM), and ConvNet (Conv), respectively.

Methods		Criteo		NASDAQ		MIMIC-III	
		AUROC	LogLoss	MSE	MRR	AUROC	AUPRC
Tree Models	GBDT (tree ensemble)	0.7853	0.46425	6.04 × 10${}^{-4}$	2.95 × 10${}^{-2}$	0.7836	0.4371
	sGBM (tree ensemble)	0.7889	0.46267	5.72 × 10${}^{-4}$	3.27 × 10${}^{-2}$	0.7883	0.4420
Deep Models	DFM/rLSTM/Conv	0.8004	0.45039	3.88 × 10${}^{-4}$	4.13 × 10${}^{-2}$	0.8728	0.5327
T&N Fusion	W&D (DAG pattern)	0.7970	0.45942	4.60 × 10${}^{-4}$	3.92 × 10${}^{-2}$	0.8783	0.5351
	GBDT-[DNNs] (T-N)	0.8136	0.44695	3.43 × 10${}^{-4}$	4.25 × 10${}^{-2}$	0.8949	0.5482
	JSDF-[DNNs] (T-N)	0.8168	0.44237	3.27 × 10${}^{-4}$	4.43 × 10${}^{-2}$	0.9015	0.5503
	[DNNs]-sGBM (N-T)	0.7958	0.46041	4.24 × 10${}^{-4}$	3.53 × 10${}^{-2}$	0.8689	0.5217
Proposed TNT	GBDT-[DNNs]-sGBM	0.8044	0.45733	3.78 × 10${}^{-4}$	4.18 × 10${}^{-2}$	0.8694	0.5410
	GBDT-[DNNs]-dGBDT	0.8079	0.44980	3.64 × 10${}^{-4}$	4.23 × 10${}^{-2}$	0.8916	0.5425
	JSDF-[DNNs]-dGBDT	0.8095	0.44887	3.51 × 10${}^{-4}$	4.29 × 10${}^{-2}$	0.8988	0.5433
